# Semi-Annual Meeting of the Erie County Medical Society

**Published:** 1881-07

**Authors:** 


### Society Reports.

### SEMI-ANNUAL MEETING OF THE ERIE COUNTY MEDICAL SOCIETY.

The semi-annual meeting of the Erie County Medical Society was held at the college building Tuesday forenoon, June 14, 1881. The attendance was unusually large. In the absence of the President, Dr. Hauenstein, Dr. T. M. Johnson, the Vice-President, occupied the chair. Dr. A. M. Barker, the Secretary, was at his post.

The committee on membership recommended the following for membership in the society: Drs. J. B. Coakley, J. Stone Armstrong, Wm. F. Granger, Judson B. Andrews, Benj. H. Grove, Frederick Peterson, Franklin Burt, Alexander S. Hancock, Samuel H. Warren, Samuel L. Atwater, all of Buffalo, and N. H. Jackson, of Springville.

Action in regard to the application of Dr. J. D. Bonner was deferred until the annual meeting in January.

The following applications for membership were received and referred to the committee on membership: Drs. C. L. Daniels, Mary E. Runner, Edward H. Clark, Ed. H. Ballow, Jno. A. Hoffmeyer, Irving M. Snow and N. F. Kiefer.

Dr. Storck, chairman of the Board of Censors, presented the following

### REPORT OF THE BOARD OF CENSORS.

*To the Erie County Medical Society:*

Your censors would respectfully report: At the annual meeting of the Society, held on the 15th of January, 1881, the Board of Censors was authorized and directed to furnish the District Attorney the names of all persons known to the Board as practicing medicine in this county in violation of the new medical law of 1880, and to urge their prosecution.

In accordance with the opinion of District Attorney Hatch and other legal advisers, the Board of Censors of a legally authorized medical society has the right, and it is its imperative duty to look into all violations of the laws of the state governing the practice as well as the study of medicine, and to bring such violations to the notice of a proper tribunal.

The new medical law passed in 1880, entitled, "An act to regulate the licensing of physicians and surgeons," has been amended by the present Legislature by an act passed May 2d, 1881, which provides as follows: "Section 1. Any person who was duly authorized to practice physic or surgery in this State and entitled to register in the office of any County Clerk in any county of this State, where such person was practicing or intending to practice physic or surgery, under and according to the provisions of (the law above referred to of 1880,) and who shall not have registered as required by the provisions of said chapter, shall have until the first day of October, 1881, in which to register as prescribed by section 1 of said act.

"Section 2. Any person who shall comply with the provisions of this act shall not be liable in any manner to the penalties prescribed by section 3 (of the Law of 1880). This section shall not apply to a person who shall have practiced physic or surgery under cover of a diploma illegally obtained, but such person shall be liable to the penalties prescribed by section 3, above mentioned."

The members of your Board, after due consideration, have come to the conclusion: That, according to the provisions of this amendment no action seems to be necessary, and no steps can be taken to prosecute any person who has not registered in accordance with the medical law of 1880, until after October 1st, 1881, and that we deem it a useless task to prosecute any one practicing "under cover of a diploma illegally obtained," or in violation of the law above referred to, as long as institutions are tolerated in this State that can assume the title "medical college," by virtue of a charter obtained under the obsolete law, entitled "An act for the organization and incorporation of benevolent, charitable, scientific and missionary *societies*, passed April 12th, 1848; whereby in our opinion the legal restrictions pertaining to the founding of medical colleges have been evaded, and also insufficient guarantee is given against the graduation of unworthy candidates.

The graduates of these institutions become legally authorized practitioners, with the right to register and to become members of any legally constituted medical society, and as such they are beyond the reach of the Board of Censors.

The members of the Board, therefore, have decided to defer further action in the execution of the new law until the legality of these questionably obtained diplomas is determined.

In order to test this question we have applied, through the well known legal advisers, Messrs Lewis & Rice, to the Attorney-General of the State for an order to bring the case of the College of Physicians and Surgeons of this city before the proper Court. This order having been granted, the question of the legality of the charter of this institution will soon be argued.

In order to meet the necessary expenses of this suit we would recommend the adoption of the following:

*Resolved*, That the Treasurer of this Society be and the same is hereby authorized and directed to pay upon the order of the Chairman of the Board of Censors all bills incurred in the prosecution of the above action.

The Board of Censors would further recommend that the Secretary of the Society be directed to notify all members to send the names of persons known to them to practice medicine in this county in violation of the new medical law, to the Board Also report the names of druggists who are known to prescribe illegally and deal out medicines to patients in their stores.

We also recommend the adoption of the following resolution :

*Resolved*, That the Erie County Medical Society request the State Board of Health to submit to the next Legislature a draft of a bill to provide for a State Board of Medical Examiners.

EDWARD STORCK,
A H. BRIGGS,
H R. HOPKINS,
WM. C. PHELPS,
P. W VAN PEYMA,
Board of Censors

Dr. Storck also read the following

REPORT OF THE SPECIAL COMMITTEE.

Your Committee, appointed at the annual meeting of the Erie County Medical Society, to submit a draft of a bill "To create a Board of Health for the City of Buffalo, composed in part of medical men," and petition the Common Council to endorse the bill and submit it to the Legislature, would respectfully report:

The Committee has memorialized the Common Council on the propriety and necessity of having a Medical Board of Health in this city, and submitted a draft of a bill. The subject matter was referred to the Committee on Sanitary Measures. After some changes having been made, the Committee unanimously agreed to it, and reported it back with its recommendation. The Common Council, however, refused to receive the report of the Committee, and postponed its consideration indefinitely The reason given for this action was that the medical profession in this city was not united on the proposed change in the Board of Health, and, strange as it may seem, the strongest opposition came from members of this Society, for reasons best known to them.

Without the approval of the Common Council, and with an opposition from our own ranks, your Committee did not deem it advisable to have the bill introduced in the present legislature, as it would probably have met there with defeat. We therefore decided to defer further action for the time being

The fact that Buffalo is the only large city in the Union that has a Board of Health not in charge of medical men, is certainly no credit to the medical societies of this city and county. Every sensible citizen will admit that the construction of our Board should be changed A Board composed of laymen, who hold the positions as ex officio members, are inefficient; in time of our epidemic, like small pox, they are totally powerless, as past experience has fully shown Questions of sanitary science and sanitary measures should be left to professional men, and to them alone should be entrusted the responsibility of the management of our health department.

The manner in which appointments are made for positions of district physicians and other employes of the Board of Health, is particularly faulty. The public press has stigmatized it as "a disgraceful scramble over the petty offices;" it has proven an annoyance annually to the members of the Board and the Common Council Applicants for these places, with a beggarly salary of a few hundred dollars, should not be obliged to become ward politicians, and sacrifice their self respect and professional honor to obtain that boon. These appointments should be made on personal claims and professional merits only, irrespective of party politics, and the appointing power should belong to the Board of Health alone if composed of medical men.

Your Committee would recommend that the bill as agreed upon by the Committee on Sanitary Measures of the Common Council, "To create a Board of Health for the city of Buffalo, composed in part of medical men," be submitted to the next State Legislature by this society, and the Senators and Members of Assembly from the District be requested to procure its passage.

BUFFALO, June 14, 1881.

EDWARD STORCK,
S F. MIXER,
J. C. GREENE,
L. P. DAYTON,
A. H. BRIGGS,
Committee.

